# Ameliorative Effects of Korean-Red-Ginseng-Derived Polysaccharide on Antibiotic-Associated Diarrhea

**DOI:** 10.3390/polym16020231

**Published:** 2024-01-14

**Authors:** Su Ji Min, Hiyoung Kim, Noriko Yambe, Myoung-Sook Shin

**Affiliations:** 1 College of Korean Medicine, Gachon University, Seongnam-si 13120, Republic of Korea; saturn13@naver.com (S.J.M.); norikoy@gachon.ac.kr (N.Y.); 2 Department of Biomedical Science and Engineering, Konkuk University, Seoul 05029, Republic of Korea; reihyoung@konkuk.ac.kr

**Keywords:** Korean-red-ginseng polysaccharide, antibiotic-associated diarrhea, short-chain fatty acid, microbiome

## Abstract

This study evaluated the ameliorative effects of Korean-red-ginseng-derived polysaccharide (KRG-P) on antibiotic-associated diarrhea (AAD) induced by administering lincomycin in mice. Changes of intestinal barrier proteins, the intestinal microbiome and short-chain fatty acid (SCFA) contents were investigated. Lincomycin was orally administered for 9 days to induce diarrhea; subsequently, 100 mg/kg and 300 mg/kg of KRG-P were administered orally for 12 days. The diarrhea was observed in the AAD group; further KRG-P administration improved the diarrhea. Analysis of changes in the intestinal microbial flora of the mice revealed that the harmful bacterial flora (such as Proteobacteria) were increased in the AAD group, whereas beneficial bacterial flora (such as Firmicutes) were decreased. However, KRG-P administration resulted in decreased Proteobacteria and increased Firmicutes, supporting the improvement of the microbial flora imbalance caused by AAD. Moreover, an analysis of the SCFAs (acetic acid, propionic acid, and butylic acid) in the caecum revealed that SCFAs’ contents in the AAD group were substantially reduced but tended to increase upon KRG-P administration. Based on these results, KRG-P, which is primarily composed of carbohydrates can improve lincomycin-induced diarrhea, likely owing to the recovery of SCFA content by improving the intestinal microbial imbalance and intestinal barrier proteins.

## 1. Introduction

Antibiotics have revolutionized modern medicine by providing effective treatments for bacterial infections, significantly reducing morbidity and mortality rates. The widespread use of antibiotics has played a pivotal role in managing various bacterial illnesses and preventing the spread of infectious diseases. Despite their significant contributions to public health, the extensive and sometimes indiscriminate use of antibiotics has led to the emergence of several challenges, including antibiotic-associated diarrhea (AAD). This side effect, resulting from alterations in the gut microbiota due to antibiotic therapy, poses a considerable clinical concern.

AAD can lead to severe complications, including dehydration, hypotension, sepsis, colonic perforation, and, in extreme cases, death [1]. The main etiology of AAD is that antibiotics cause an imbalance in the intestinal flora or change the short-chain fatty acid (SCFA) content, resulting in an increase in unabsorbed carbohydrates. An imbalance in intestinal microorganisms has been reported to increase intestinal sensitivity, cause obesity owing to systemic metabolic disorders, and affect homeostasis in the human body, resulting in allergic and autoimmune diseases [2,3,4,5].

When classifying microorganisms isolated from the intestines of healthy adults at the phylum level (such as Firmicutes, Bacteroidetes, Actinobacteria, Fusobacteria, Proteobacteria, Verrucomicrobia, and Cyanobacteria), Firmicutes and Bacteroidetes have been reported to dominate, with the remaining phyla accounting for less than 10% [6]. Therefore, it can be said that the ratio of Firmicutes and Bacteroidetes is closely related to the composition of human intestinal microorganisms. Additionally, they inhibit the growth of pathogens through the synthesis of antibacterial substances and host immune response [7]. Carbohydrates that resist digestion in the small intestine, such as starch or dietary fiber, undergo fermentation by intestinal microorganisms in the large intestine to produce SCFAs like acetate, butyrate, and propionate [8]. These SCFAs act as direct energy sources for the intestinal epithelium. Acetate is the primary substrate for cholesterol synthesis in the colon; butyrate is used as an energy resource in the colonic epithelium, and proportionally reduces the amount of cholesterol [9]. Intestinal microorganisms play a crucial role, as well, in maintaining the structural integrity of the digestive tract, influencing the development and differentiation of intestinal epithelial cells, promoting intestinal villi development, and contributing to capillary formation, thereby ensuring the structure and function of the intestinal mucosal layer.

Korean ginseng (*Panax ginseng* C. A. Meyer) comprises about 60–70% of starch along with key chemical components such as polyacetylenes, aromatic compounds, acidic peptides, and ginsenosides, which constitute the main pharmacological components of ginseng [10,11,12]. Furthermore, the high-temperature processing of ginseng, resulting in red ginseng, is recognized for its efficacy in cancer prevention, blood pressure reduction, antioxidative properties, and as an antithrombotic agent [13,14,15,16,17,18,19]. Previous research indicates that polysaccharides derived from natural sources exhibit a diverse range of activities, including anti-inflammatory, anti-complement, anti-tumor, antioxidant, blood-sugar-lowering, immune regulation, and phagocytosis-enhancing activities [20,21,22,23,24,25]. Notably, their low toxicity and minimal side effects make them noteworthy candidates as functional materials and potential substitutes for synthetic drugs in the food and pharmaceutical industries [26]. Ginseng polysaccharides have been reported to exhibit protective effects on gastrointestinal epithelial cells in mice, demonstrating utility in the treatment of conditions such as gastric ulcers and colitis through their anti-inflammatory activities [27]. Moreover, the acidic polysaccharides of red ginseng possess anticancer effects [28] and can regulate the immune response, including the activation of macrophages [29,30].

In a recent report, we highlighted the immunomodulatory potential of a polysaccharide derived from Korean red ginseng, KRG-P, showcasing its ability to regulate intestinal immunity through Peyer’s patches and its potential in safeguarding against intestinal infections by promoting IgA secretion [31]. Building on these findings, the current study aimed to assess the effectiveness of KRG-P administration in alleviating diarrhea using an AAD-induced model. Additionally, we examined its impact on fortifying the intestinal barrier, as well as its role in modulating alterations in intestinal microorganisms and SCFAs.

## 2. Materials and Methods

### 2.1. Fourier Transform Infrared Spectroscopy Analysis

The Fourier transform infrared spectroscopy (FT-IR) spectra of a KRG-P were obtained using a VERTEX80v spectrophotometer (Bruker, Bremen, Germany). The KRG-P was blended with KBr powder at a mass ratio of 3:1 and compressed into pellets. The FT-IR spectra were recorded in the range of 600–4000 cm^−1^.

### 2.2. Nuclear Magnetic Resonance Analysis

The lyophilized KRG-P was dissolved in deuterium oxide (D_2_O) (D 99.90%, Cambridge Isotope Laboratories, Tewksbury, MA, USA). The 1D (^1^H and ^13^C) nuclear magnetic resonance (NMR) spectra were acquired using an 850 MHz Bruker Avance III HD (Bruker, Ettlingen, Germany).

### 2.3. Preparation of the Polysaccharide Fraction from Red Ginseng

KRG-P was prepared as previously described [31]. KRG extract, recognized by the Ministry of Food and Drug Safety (formerly known as the Korea Food and Drug Administration) as a functional health food [24], was provided by the Korea Ginseng Corporation (Seoul, Republic of Korea). KRG was first diluted ten times with purified water and then four times with ethanol, and the mixture was slowly stirred until the polysaccharide fraction precipitated. The precipitate was collected using centrifugation (600× *g* for 20 min), dissolved in purified water, dialyzed with Slide-A-Lyzer 20 K dialysis cassettes (molecular weight cut-off: 20,000 Da) to remove ethanol and low-molecular-weight substances, and finally lyophilized (KRG-P yield: 25%).

### 2.4. Animal and Experimental Design

**Figure 1:**
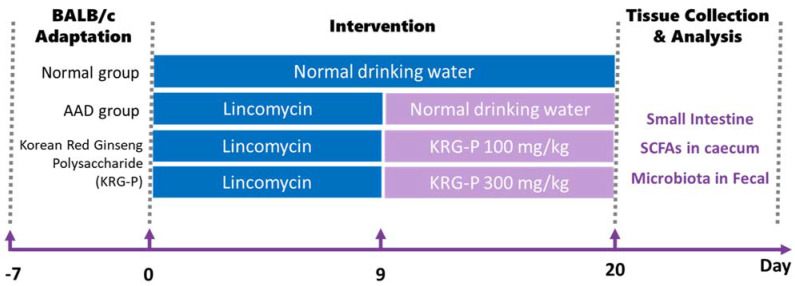
Oral administration schedule for KRG-P in the lincomycin-induced AAD mice model. AAD was induced with the administration of 3 g/kg lincomycin for 9 days. CMC solutions were administered to the normal group. The KRG-P group was orally administered 100 or 300 mg/kg of KRG-P for 12 days.

BALB/c mice (7 weeks old, female) were purchased from Orient Bio (Seongnam, Republic of Korea). After a 7-day adaptation period, the animals were assigned to the following groups: control group (normal), antibiotic-induced-diarrhea group (AAD), low-dose-treatment group (AAD + KRG-P 100 mg/kg), and high-dose-treatment group (AAD + KRG-P 300 mg/kg). Subsequently, the control group was orally administered physiological saline, and the AAD group was orally administered lincomycin (Dongkwang Pharm, Seoul, Republic of Korea) at a concentration of 3 g/kg once daily for 9 days. After the completion of lincomycin periods, both the control and AAD groups received an oral administration of sterilized 0.5% *w*/*v* methylcellulose 400 solution (CMC) (Wako, Tokyo, Japan). In addition, lincomycin was orally administered to the treatment groups for 9 days, which were then orally administered KRG-P at concentrations of 100 or 300 mg/kg for 12 days. The experimental animals were exposed to a 12 h light–dark cycle and provided ad libitum access to food and water in an environment with a temperature of 22 ± 2 °C and humidity of 50–55%. Mouse fecal scores were assigned according to the diarrhea status. At the end of the experiment, the animals were dissected, and small intestine, caecum, and feces were collected and stored at −80 °C (Figure 1). All animal experiments were conducted in accordance with the guidelines of the Institutional Animal Care and Use Committee (IACUC) of Gachon University (Approval No: GU1-2022-IA0050-00).

### 2.5. Preparation of Tissue Lysate and Immuno-Blotting

Mouse small intestine tissue was cut and stored frozen at −80 °C. After removing the tissue and thawing it on ice, it was placed in a Disposable BioMasher (TAKARA, Shiga, Japan) and ground while maintaining the ice state using RIPA buffer (including protease inhibitor, 1 mM DTT). Then, centrifugation was performed (13,000 rpm, 20 min, 4 °C), and only the supernatant was collected for protein quantification. It was diluted with RIPA buffer to a concentration of 1 mg/mL. After mixing and denaturing the sample buffer and proteins, SDS-PAGE (Mini-Protean TGX, Precast Gel, Bio-Rad, Hercules, CA, USA) was performed. After transferring the separated proteins to a PVDF membrane (Millipore, Burlington, MA, USA), phosphorylation of the proteins was detected using the antibodies provided in Table 1. The phosphorylated band of each protein was quantified using the ImageJ quantification program, graphed, and statistically analyzed.

### 2.6. Quantitative Real-Time Reverse-Transcription Polymerase Chain Reaction (qRT-PCR)

Mouse small intestinal tissue was extracted using BioMasher (Takara, Shiga, Japan) and filtered using QIAshredder (Qiagen, Hilden, Germany). The total RNA from the intestinal tissue was isolated and purified using the RNeasy Mini Kit (Qiagen) and reverse transcribed into cDNA using the RevertAid First Strand cDNA Synthesis kit (Fermentas, Waltham, MA, USA) according to the manufacturer’s protocols. qRT-PCR was performed using the Mm01228299_m1 (lysozyme) and Mm01342184_m1 (claudin1) TaqMan primer sets (Applied Biosystems, Waltham, MA, USA). The amplification conditions were determined using a QuantStudio-3 real-time PCR system (Applied Biosystems). Data were normalized to the ß-actin levels.

### 2.7. Measurement of IgA in Fecal Matter of Mice Orally Administered KRG-P

In the AAD model, the feces of mice orally administered KRG-P were collected and stored at −80 °C. To measure fecal IgA, the weight of the feces was measured, and phosphate-buffered saline (PBS, pH 7.2) was added and suspended to achieve a concentration of 1 mg/mL. The samples were thoroughly mixed using a stirrer and left on ice for 20 min. They were centrifuged at 5000 rpm for 20 min at 4 °C. The supernatant was obtained, and the IgA levels were measured. The IgA levels were measured using an ELISA kit, according to the manufacturer’s protocol (Invitrogen, Carlsbad, CA, USA).

### 2.8. 16S rRNA Sequencing of Fecal Matter

After inducing AAD, KRG-P was administered orally, and mouse fecal matter was collected on day 12 and stored at −80 °C. To identify the microorganisms in mouse feces, 16S rRNA sequencing was performed by Macrogen, Inc. (Seoul, Republic of Korea).

### 2.9. Determination of SCFA Content

To evaluate the SCFA content of the mouse caecum, it was ground to a concentration of 1 g/mL in an 80% methanol solution using a BioMasher (TaKaRa, Shiga, Japan). Centrifugation was performed at 13,000 rpm for 10 min at 4 °C, and the supernatant was filtered using 0.45 μm syringe filters from ADVANTEC. The analysis was performed using a flame ionization detector (Hewlett Packard, Palo Alto, CA, USA) and a GC column (DB-FFAP 123-3253, 50 mm × 0.32 mm × 0.50 μm, Agilent Technologies, Inc., Santa Clara, CA, USA). SCFA concentrations were quantified using acetic acid, propionic acid, and butyric acid as standards. The SCFA content in the cecum was determined using a calibration curve based on the corresponding standards. Acetic acid, propionic acid, and butyric acid were purchased from Sigma-Aldrich (St. Louis, MO, USA).

### 2.10. Statistical Analysis

The results of three independent experiments are expressed as mean ± standard deviation (SD). All statistical analyses were performed using one-way analysis of variance (ANOVA), followed by Tukey’s post hoc test. The data are expressed as mean ± SD.

## 3. Results

### 3.1. NMR and FT-IR Analysis of KRG-P

**Figure 2:**
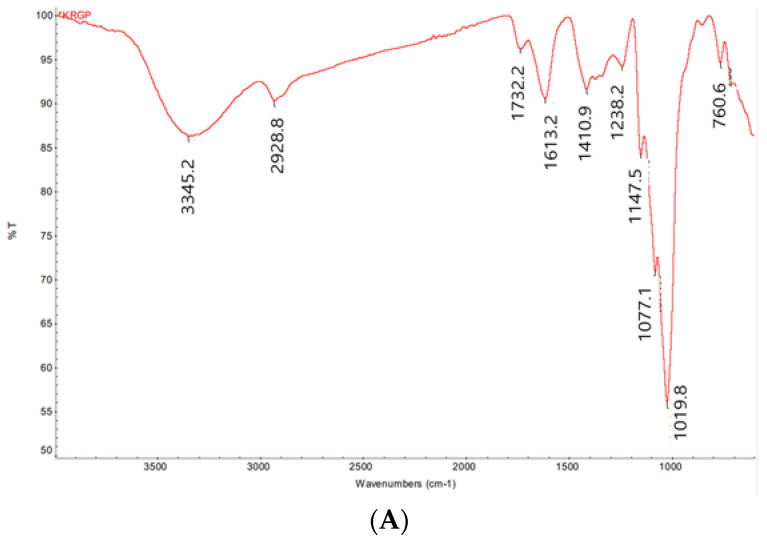

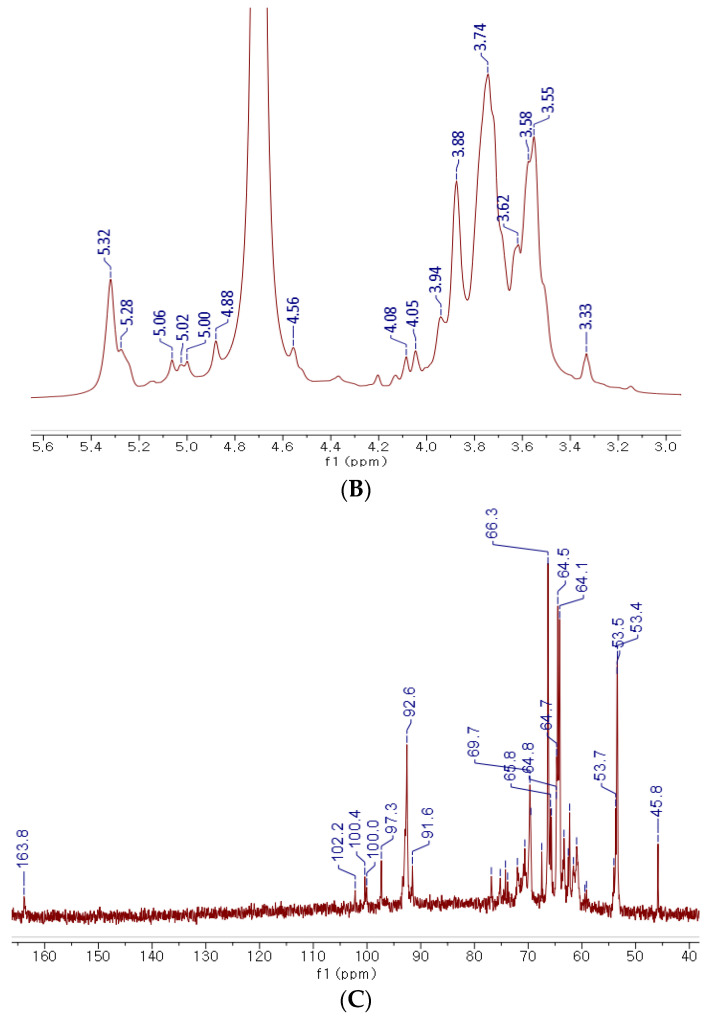
NMR and FT-IR analysis of KRG-P. FT-IR spectrum of KRG-P (**A**), ^1^H NMR spectrum of KRG-P (**B**), ^13^C NMR spectrum of KRG-P (**C**).

The chemical properties and monosaccharide composition of KRG-P were previously reported using high-performance liquid chromatography [31]. Here, we present additional spectral data to provide detailed chemical information, enabling a more comprehensive understanding of the chemical composition of KRG-P. The FT-IR spectrum of KRG-P displayed distinctive features, with hydroxyl and C-H stretching vibrations observed at 3345.2 and 2928.8 cm^−1^, respectively. The absorption peaks at 1613.2 and 1732.2 cm^−1^ were attributed to bound water and the C=O stretching vibration of the uronic acid, respectively. Furthermore, absorption peaks at 1410.9 and 1238.2 cm^−1^ indicated C-H angular vibrations of carbohydrates. Peaks at 1147.5, 1077.1, and 1019.8 cm^−1^ were associated with C-O-H and C-O-C stretching vibrations of pyran, suggesting that KRG-P is linked with α-pyranoside bonds. Additionally, the absorption peaks at 925.1 and 855.2 cm^−1^ were characteristic of α-Glcp (Figure 2A).

The ^1^H NMR spectrum of KRG-P revealed the presence of anomeric proton signals within the range of δ5.32 to δ4.56 ppm derived from monosaccharides (Figure 2B). Strong signals falling within δ3.33 to δ4.20 ppm corresponded to H-2 to H-6 protons, indicating the presence of CH-O and CH_2_-O groups in the carbohydrate structure. To gain further insights into the structure of KRG-P, ^13^C NMR spectroscopy was performed. The signal at δ170.85 ppm was attributed to carboxylic acid from galacturonic acid (Figure 2C). The detection of carbon chemical signals in the range of δ91.6 to δ102.2 ppm indicates the presence of anomeric carbons derived from monosaccharides such as glucose, galactose, arabinose, and rhamnose. These monosaccharides were predicted in the analysis of the monosaccharide composition of KRG-P in the previous study [31].

In summary, these findings not only align with previously reported results on the determination of KRG-P monosaccharide composition but also enhance the depth and rigor of KRG-P characterization through the incorporation of new spectroscopic data. This contributes to a more robust validation of the reported physiological characteristics.

### 3.2. Effects of KRG-P on AAD in Mice

**Figure 3:**
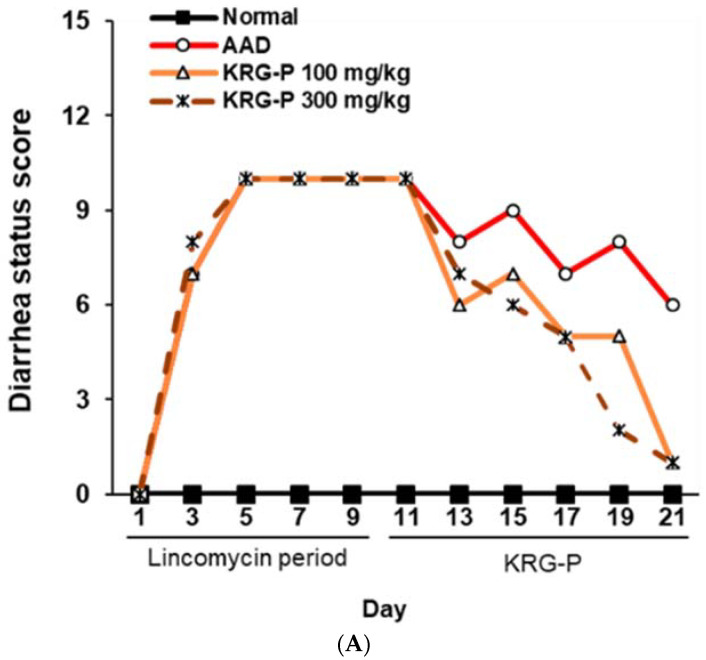

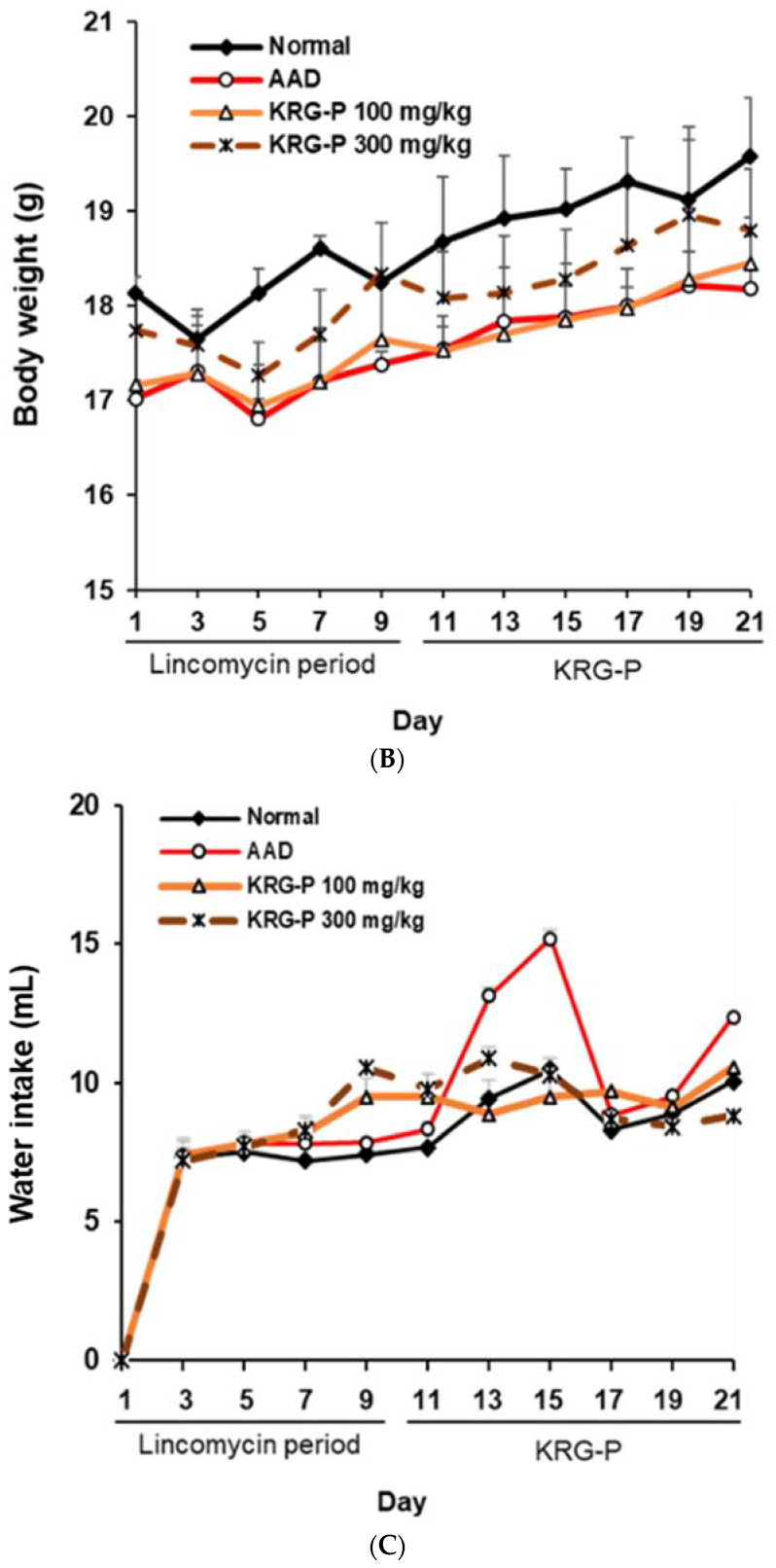
Effects of KRG-P on total diarrhea-status scores, average body weight, and water intake in the AAD mice model. The diarrhea status scores (**A**), body weight (**B**), and water intake (**C**) were assessed once every 2 days during experiment.

To evaluate the effectiveness of KRG-P in improving diarrhea in the AAD mouse model, diarrhea was induced using lincomycin hydrochloride. Subsequently, the body weight, diarrhea-status score, and water intake of the mice were measured. The severity of diarrhea was evaluated according to the criteria for diarrhea-status evaluation, as shown in Table 2. In the AAD group treated with lincomycin hydrochloride alone, the diarrhea-status score and water intake increased, and weight gain decreased compared to the normal group (Figure 3). KRG-P administration (100 mg/kg and 300 mg/kg) significantly lowered the diarrhea score compared to the AAD group. Analysis of water intake after lincomycin administration showed that the amount of water consumed in the KRG-P-administration group was similar to that in the normal group. The weight-gain rate was decreased following lincomycin administration; however, we found that the weight-gain rate was improved in the group that were administered a high concentration of KRG-P (Figure 3). These results confirmed that compared to the AAD group, the KRG-P group recovered their lost body weight and exhibited improvement in diarrhea and normalized water consumption.

### 3.3. Effects of KRG-P on Lysozyme and Claudin-1 Expression in AAD-Induced Mice

**Figure 4:**
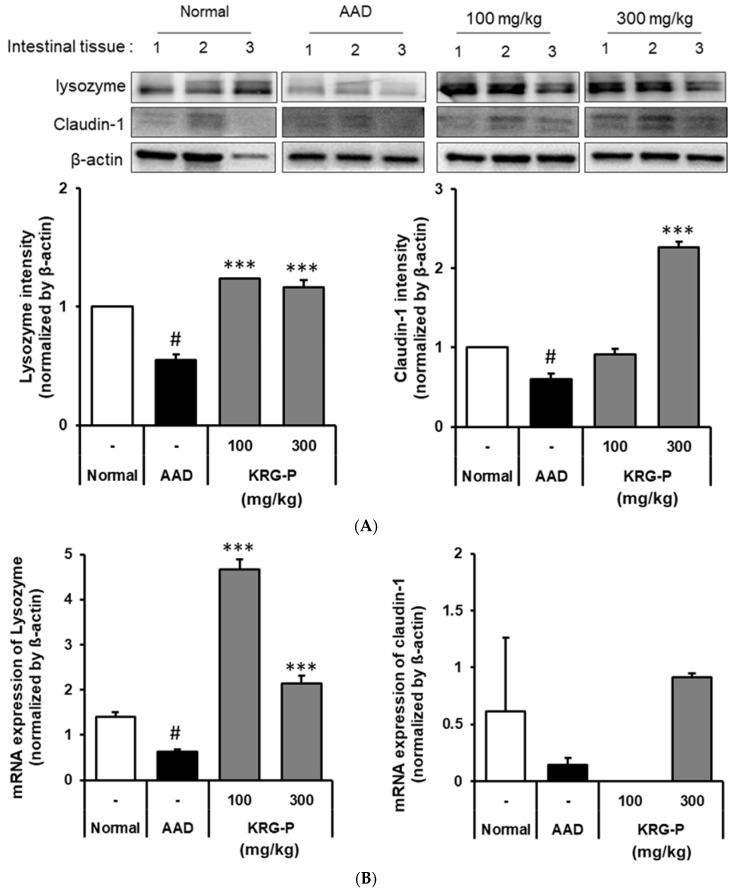
Effects of KRG-P on lysozyme and claudin-1 expressions in the AAD mice model. (**A**) The lysozyme and claudin-1 expressions were validated using the Image J software. (**B**) The mRNA expressions of lysozyme and claudin-1 were determined using RT-qPCR. Statistical analysis was performed using one-way ANOVA, followed by Tukey’s post hoc test using Prism 5. Data are presented as the mean ± standard deviation (SD) of triplicate experiments. # *p* < 0.0001 vs. Normal group; *** *p* < 0.0001 vs. AAD group.

We analyzed the effects of KRG-P on lysozyme and claudin-1 expression in the small intestines of AAD-induced mice (Figure 4A). The lysozyme protein expression was significantly decreased in the AAD-induced group compared to that in the normal group. In the KRG-P (100 mg/kg and 300 mg/kg)-administration groups, the expression was significantly increased compared to that in the AAD group.

Moreover, there was a tendency for the claudin-1 protein expression to decrease in the AAD group compared to the normal group. In the KRG-P-administered group, the decreased expression of claudin-1 was confirmed to improve in a concentration-dependent manner. These results confirmed that KRG-P administration restored the expression of lysozyme and claudin-1, which was reduced owing to AAD.

Furthermore, the mRNA expression levels of lysozyme and claudin-1 were analyzed (Figure 4B). We found that the lysozyme mRNA expression decreased in the AAD group compared to that in the normal group. In contrast, the lysozyme mRNA expression increased in the KRG-P-administered group. The mRNA expression level of claudin-1, which decreased in the AAD group, tended to increase in the KRG-P-administration group (300 mg/kg).

These results confirmed that KRG-P administration increased the mRNA expression levels of claudin-1 and lysozyme, which were reduced in the lincomycin-induced AAD model, suggesting that KRG-P helped maintain intestinal homeostasis.

### 3.4. Effects of KRG-P on IgA Secretion in AAD-Induced Mice

We have previously reported that KRG-P administration increases intestinal IgA production [31]. The Peyer’s patch is the center of IgA secretion in the intestinal tract, and it can be expected that fecal matter contains a significant amount of IgA secreted in the intestine. Therefore, in the AAD model, KRG-P was orally administered at 100 and 300 mg/kg, and the residual IgA present in mouse feces was assessed.

**Figure 5:**
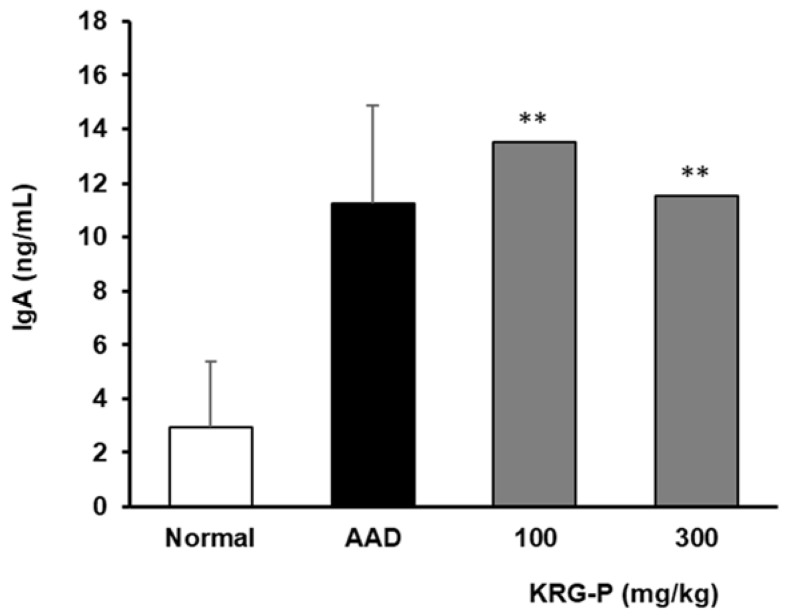
IgA levels in the fecal matter of mice administered KRG-P in the AAD mice model. IgA secretion was determined using mouse ELISA kits. Statistical analysis was performed using one-way ANOVA, followed by Tukey’s post hoc test using Prism 5. The data are presented as the mean ± SD (n = 5). ** *p* < 0.001 compared to the normal group.

We found that the IgA content in the feces significantly increased in the group administered with KRG-P (Figure 5). Collectively, our results showed that KRG-P administration may have activated Peyer’s patches and promoted IgA secretion in the intestinal tissue in the AAD model. KRG-P may also be effective in removing foreign antigens introduced into the intestine.

### 3.5. Analysis of the Changes in the Intestinal Microflora of Mice in the KRG-P-Administered AAD Model

Changes in the intestinal microflora are influenced by various factors, such as the environment, diet, and antibiotic use [32]. Additionally, microbial imbalance causes diseases such as inflammatory bowel disease and irritable bowel syndrome [33]. However, increasing beneficial intestinal bacteria and decreasing pathogenic microorganisms that interact with the host can positively affect the prevention and treatment of various intestinal diseases [34].

**Figure 6:**
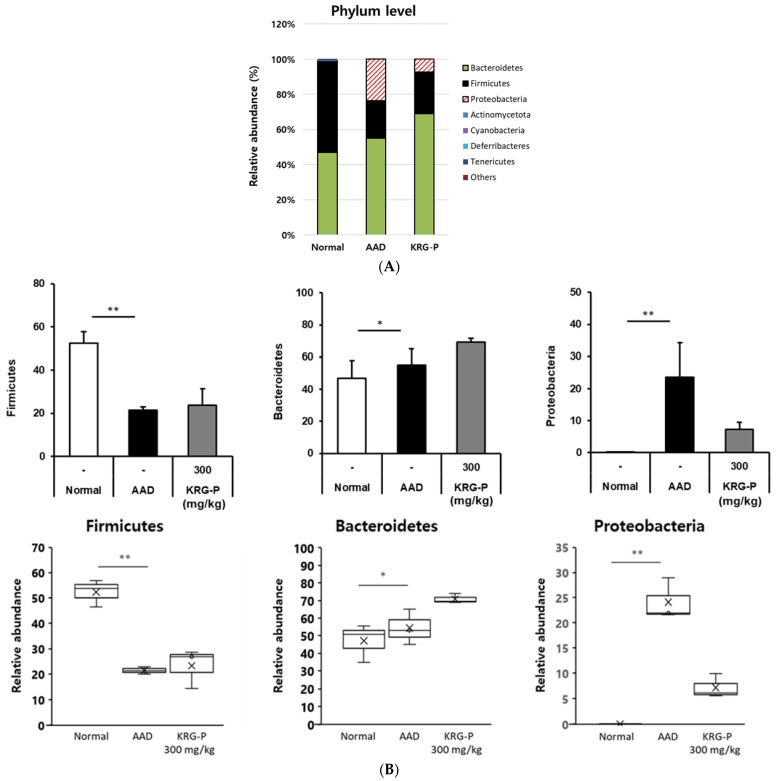
Relative abundance of the bacterial communities. (**A**) Phyla level. (**B**) General level. (n = 3). ** *p* < 0.001, * *p* < 0.05.

Therefore, in this study, we analyzed the effects of KRG-P on the intestinal microbial community in the AAD model. Using 16S rRNA classification-based analysis of mouse feces, microbial differences were confirmed at the phylum level in the normal, AAD, and KRG-P (300 mg/kg) groups (Figure 6). In the normal, AAD, and KRG-P groups, approximately 52.04%, 21.39%, and 23.71% of the microorganisms, respectively, belonged to the Firmicutes phylum. Approximately 46.92%, 55.03%, and 69.04% of the microorganisms, respectively, belonged to the Bacteroidetes phylum. Moreover, approximately 0.03%, 23.58%, and 7.25% of the microorganisms, respectively, belonged to the Proteobacteria phylum. Analysis of intestinal microorganisms at the phylum level showed that Firmicutes and Bacteroidetes accounted for more than 70% of the total intestinal microorganisms in all groups.

In the AAD model, Firmicutes and Bacteroidetes’ abundance, which decreased owing to AAD was increased following KRG-P administration. Proteobacteria abundance, which increased in the AAD group, tended to decrease after KRG-P administration. This indicated that KRG-P contributed to the regulation of the composition and diversity of intestinal microorganisms. In addition, by reducing the abundance of the harmful intestinal bacteria increased by AAD, KRG-P may serve as a new therapeutic substance that can not only improve diarrhea caused by antibiotic use but also resolve the intestinal microbial imbalance.

### 3.6. Analysis of SCFAs in the Cecum of Mice in the KRG-P-Administered AAD Model

**Figure 7:**
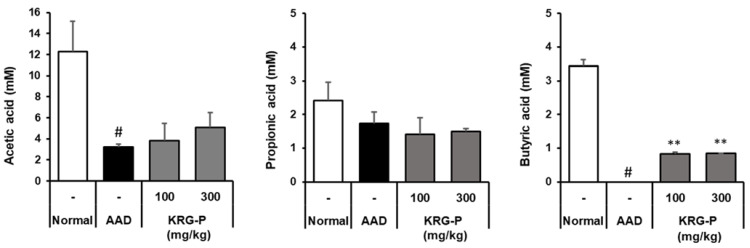
Effects of KRG-P on short-chain fatty acids in the cecum of the lincomycin-induced AAD mice model. Acetic acid, propionic acid, and butyric acid levels in the mouse cecum were determined using gas chromatography. Data are presented as the mean ± standard deviation (SD) of triplicate experiments. # *p* < 0.0001 vs. Normal group; ** *p* < 0.001 vs. AAD group.

The main SCFAs produced by intestinal microbial fermentation are acetic acid, propionic acid, and butyric acid. SCFAs are absorbed by colonic epithelial cells and stimulate water and electrolyte absorption to relieve diarrheal symptoms. The effect of KRG-P on SCFAs in the mouse cecum was analyzed in the AAD model (Figure 7). We observed that the acetic acid content in the AAD-induced group was significantly lower than that in the normal group. In contrast, when KRG-P was administered at 100 or 300 mg/kg, the decrease caused by AAD induction recovered in a concentration-dependent manner. Propionic acid levels were lower in the AAD group than in the normal group; however, there was no tendency toward recovery following KRG-P administration. Regarding butyric acid, butyric acid content was not detected in the AAD group but tended to increase in the KRG-P-treated group.

It has been reported that butyric acid can induce regulatory T (Treg) cells in the colon and suppress inflammation and allergic reactions [35]. Our results showed that KRG-P administration restored the reduced content of acetic acid and butyric acid in the AAD model and acted as an important factor in regulating the intestinal environment. Therefore, KRG-P may be effective in the prevention and treatment of intestinal diseases.

## 4. Discussion

In modern medicine, the inappropriate use of antibiotics is associated with various side effects [36]. Diarrhea caused by excessive antibiotic administration changes the structure and function of the intestinal microflora [37]. Studies have reported that medicinal plants induce physiological changes by regulating intestinal microflora [3]. Moreover, our previous study reported that KRG-P can regulate intestinal immunity and protect against intestinal infections [31]. In this study, we investigated the effects of KRG-P on the maintenance of intestinal homeostasis in an AAD-induced mouse model.

Most foods, herbal medicines, and other medicines enter the human body through ingestion. Therefore, the immune responses in the intestinal tract, where they are first absorbed by the body, and intestinal immunity, including the Peyer’s patches, are very important. When cells within Peyer’s patches are activated, cytokines are produced, which activate B lymphocytes to differentiate into plasma cells and induce IgA secretion [38]. IgA plays a central role in the intestinal immune system, as it inhibits the attachment of various harmful substances and pathogenic microorganisms in the intestinal tract to the mucosal epithelium and possesses antibody activities against bacteria and viruses entering the intestinal tract. Additionally, regarding the IgA content present in feces, we found that it significantly increased in the KRG-P-administered group (Figure 5). These results suggest that KRG-P can increase IgA secretion and activate intestinal immune function. Tight junction proteins, claudin-1 is important in the intestinal defense barrier and is a signal transduction mediator in various diseases. Lysozyme is a 1,4-β-N-acetylmuramidase that kills cells by cleaving the glycosidic binding site of peptidoglycan, a component of the bacterial cell wall. In addition, hydrolyzed products by lysozymes have been reported to improve IgA secretion, macrophage activity, and the removal of pathogenic bacteria [39]. The effects of KRG-P on lysozymes and claudin-1 expression in the small intestine were analyzed using the AAD model. Lysozyme expression significantly decreased in the AAD group. It significantly increased in the KRG-P treatment compared to that in the AAD group at all concentrations. Additionally, the expression of claudin-1 tended to decrease in the AAD group. In the KRG-P-treated group, the decreased expression of claudin-1 improved. These results confirmed that KRG-P administration restored the expression of lysozyme and claudin-1, which were reduced owing to AAD (Figure 4A). Additionally, claudin-1 and lysozyme mRNA expression showed a similar tendency to that of their protein expression (Figure 4B). Therefore, KRG-P administration may be effective in strengthening the intestinal barrier.

The intestinal microflora represents the environment in which microorganisms survive, therefore, the condition of the intestine. Among the phyla of intestinal microorganism classification, Bacteroidetes and Firmicutes are used as indicators to determine the health status of the human body (host) depending on the ratio of the two phyla. The distribution of intestinal microorganisms in the normal, AAD, and KRG-P (300 mg/kg) groups was analyzed at the phylum level (Figure 6). Analysis of the intestinal microorganisms at the phylum level revealed that the abundance of Firmicutes decreased and that Proteobacteria increased in the AAD group. In contrast, following KRG-P administration, the abundance of Firmicutes increased, and that of Proteobacteria decreased, similar to that in the normal group (Figure 6B). According to previous studies, when infected with *C. difficile*, the abundance of Firmicutes and Bacteroidetes is reduced, and that of Proteobacteria is increased [40]. The Proteobacteria phylum comprises many microorganisms associated with inflammation [41]. In summary, KRG-P administration can restore the imbalance of intestinal microorganisms by increasing Firmicutes abundance, which was decreased owing to AAD, and can reduce Proteobacteria abundance, which was increased owing to AAD.

The reduced diversity of intestinal microorganisms reduces the production of SCFAs, which are major metabolites of microorganisms [42]. Owing to differences between species of intestinal microorganisms, their composition and the types of SCFAs produced vary depending on the type of dietary fiber consumed. Among the SCFAs, acetic acid reduces the intestinal pH and reduces the number of harmful bacteria, as their growth is inhibited under acidic conditions [43]. Owing to this effect, SCFAs reduce the incidence of irritable bowel syndrome and inflammatory bowel disease. Butyric acid is absorbed by colonic epithelial cells and increases the expression of claudin, a transmembrane protein, strengthening the tight junctions of intestinal epithelial cells [44] and alleviating inflammatory bowel disease. Additionally, butyrate is effective in alleviating diarrheal symptoms by absorbing moisture and ions in the intestines [45]. In the AAD model, the effect of KRG-P on SCFAs production in the mouse caecum was analyzed (Figure 7). We found that the acetic acid content in the AAD-induced group decreased compared to that in the normal group and recovered with KRG-P administration. Butyric acid content was not detected in the AAD group but tended to increase in the KRG-P-administered group. Overall, it was observed that KRG-P was effective in restoring the balance of altered intestinal microbial flora and SCFAs in the AAD-induced model. Therefore, KRG-P can alleviate the symptoms of antibiotic-related diarrhea and can be effective in preventing and treating intestinal metabolic diseases.

## 5. Conclusions

The use of antibiotics is essential in modern medicine, and yet excessive usage leads to side effects such as diarrhea. Recently, natural resources, which are relatively safe and have fewer side effects, have emerged to overcome the drawbacks of synthetic drugs. Particularly, Korean red ginseng is a traditional natural remedy in East Asia. Recent reports highlight its immunomodulatory, anticancer, and cancer-inhibiting properties derived from the polysaccharides isolated from Korean red ginseng (KRG).

Building upon our prior study demonstrated the intestinal immune-regulatory activity and intestinal barrier enhancement in mice upon oral administration of KRG-derived polysaccharides (KRG-P), this study aimed to analyze the potential of KRG-P in ameliorating antibiotic-associated diarrhea. Results confirmed that KRG-P administration restored microbial imbalance within the gut, exerting beneficial effects on the gut environment. Furthermore, it increased IgA and GM-CSF in mouse Peyer’s patches and enhanced the expression of lysozyme and claudin-1 in the mouse colon. This signifies the regulation of intestinal immune activity and mucosal integrity, potentially improving antibiotic-associated diarrhea. By reducing intestinal pathogenic microbes and increasing SCFAs, this approach alleviates gut disorders and mitigates antibiotics-associated diarrhea, proposing a natural-resource-based therapeutic strategy. The observed positive effects underscore the promising role of KRG-P in promoting gut health, offering a valuable avenue for further exploration in addressing gastrointestinal challenges associated with antibiotic use.

## Figures and Tables

**Table 1:** List of antibodies used for the Western blot analysis.

Antibodies and Reagents	Source	Catalog Number
Lysozyme	Abcam	ab108508
Claudin-1	Abcam	ab180158
Anti-rabbit IgG	Cell Signaling Technology	#7074
β-actin	Cell Signaling Technology	#4967

**Table 2:** Scoring criteria for lincomycin-induced antibiotic-associated diarrhea in BALB/c mice.

Scores	Diarrhea Status
0	Normal (no diarrhea)
1	Loose, light color, and nonstick perianal stool status, generally good mental state
2	Adhesion to stool at the anus, mental depression, no appetite for food, weight loss

## Data Availability

Data are contained within the article.
